# Trust based attachment

**DOI:** 10.1371/journal.pone.0288142

**Published:** 2023-08-23

**Authors:** Julian Kates-Harbeck, Martin Nowak

**Affiliations:** 1 Department of Physics, Harvard University, Cambridge, MA, United States of America; 2 Department of Mathematics, Harvard University, Cambridge, MA, United States of America; 3 Department of Organismic and Evolutionary Biology, Harvard University, Cambridge, MA, United States of America; University of Zaragoza, SPAIN

## Abstract

In social systems subject to indirect reciprocity, a positive reputation is key for increasing one’s likelihood of future positive interactions [1–13]. The flow of gossip can amplify the impact of a person’s actions on their reputation depending on how widely it spreads across the social network, which leads to a percolation problem [14]. To quantify this notion, we calculate the expected number of individuals, the “audience”, who find out about a particular interaction. For a potential donor, a larger audience constitutes higher reputational stakes, and thus a higher incentive, to perform “good” actions in line with current social norms [7, 15]. For a receiver, a larger audience therefore increases the trust that the partner will be cooperative. This idea can be used for an algorithm that generates social networks, which we call trust based attachment (TBA). TBA produces graphs that share crucial quantitative properties with real-world networks, such as high clustering, small-world behavior, and powerlaw degree distributions [16–21]. We also show that TBA can be approximated by simple friend-of-friend routines based on triadic closure, which are known to be highly effective at generating realistic social network structures [19, 22–25]. Therefore, our work provides a new justification for triadic closure in social contexts based on notions of trust, gossip, and social information spread. These factors are thus identified as potential significant influences on how humans form social ties.

## Introduction

Models of indirect reciprocity study the effect of reputational information on the actions of individuals in a group setting. They typically assume well mixed population structures [1–7, 9–12, 15]. However, because such information spreads via communication among individuals, it is affected by the structure of the social network underlying the group. A more sophisticated model of indirect reciprocity therefore might also take into account the effects of network structure [26, 27]. The spread of information on networks generally (including effects of social contagion), as well as its dependendence on global and local network features has been explored extensively [28–34]. However, the influence of network structural effects on reputational information spread specifically remains poorly studied.

While it is clear that network structure can influence reputation spread, the reverse is also true. The connections between individuals themselves can develop dynamically based on choices and incentives shaped by indirect reciprocity [26, 35–38]. In general, this leads to a complex interdependence between the spread of reputational information and network structure, potentially across multiple time scales, which poses a formidable modeling challenge. As a step towards jointly modeling reciprocity and network evolution, some papers have proposed models including information flow and link formation on networks in the context of repeated games [26, 35–38]. In these models, an individual may update links based on the past behavior of other agents in the repeated game, resulting in networks that co-evolve with individuals’ strategies. While these papers do not study directly which network properties enhance or suppress the spread of gossip, their findings that cohesive, tightly clustered networks encourage cooperative behavior [36, 37] are in line with our results. These models do not consider strategic link formation or the effects of separate time scales between network evolution and individual behaviors.

Instead of attempting to capture the full interdependence of reputational and structural effects, we propose here an intermediary step where we first identify local structural features that relate strongly to reputation spread, and then study attachment strategies for individuals interested in building cooperative relationships in the presence of indirect reciprocity. Our objective is to provide a crisper mechanistic understanding than might be possible in a co-evolving model. We aim to illuminate static network features affecting reputation spread, as well as provide a possible game theoretical justification for some of the observed (growth) properties of real networks. The two key assumptions are (i) that reputation information can spread across the local network [14, 28–34, 39] and (ii) that individuals seek to form attachments that are likely to be cooperative (either for forward looking strategic reasons or because this happens to be a winning social strategy in a repeated game setting) [35–38].

In particular, we introduce a simple model of reputation spread that considers the expected number of individuals *n*_*ij*_ learning about an actor *i*’s good deed to one of their neighbors *j*, as a function of the local network structure. The model has a single parameter *p* which represents the strength or likelihood of communication and gossip spread between individuals. We take the quantity *n*_*ij*_ to be a proxy of the strength of reputational incentives for that relationship. This motivates a strategy of attachment where individuals select for new links that increase incentives for mutually beneficial relationships. We call the resulting algorithm “trust-based attachment” (TBA). We show empirically and motivate mathematically that the simple and well-studied network formation strategy of triadic closure represents a realistic and effective heuristic for TBA in the limit of weak gossip (low *p*). Since triadic closure specifically generates network properties consistent with several features of real social networks (better even than the original TBA), we conclude that triadic closure may both be the real mechanism used in network growth, and also that it may have developed as a heuristic for a TBA-like attachment strategy. As such, we develop a link between the dynamics of indirect reciprocity and network growth.

There is an extensive literature on models of network growth that can reproduce various statistical properties [40, 41] of real world networks. Small world behavior can arise with a small number of random long-range connections in otherwise densely locally connected networks [16]. Models based on randomly connected groups of communities of various sizes and densities [22] can reproduce both clustering and degree statistics of target networks. Scale free degree distributions can emerge from preferential attachment [17], and a trade-off between popularity and similarity can accurately model additional common network properties such as their hyperbolic geometry and high local clustering [19].

While there is evidence for scale free degree distributions in real newtorks, as well as for preferential attachment-like affinities during network growth [40], it should be noted that not all networks with heavy tailed degree distributions follow true power-law behavior. Moreover, the mechanism underlying such properties can often provide more insight than their mere statistical observation [42–44]. More generally, while the above models have applicability and supporting evidence both in social and other (e.g. biological, technological, physical) contexts, the justification for their mechanisms in all those settings is not always clear. Several of these models also seemingly require global knowledge of the network (such as the degree of all nodes) for an agent to effectively implement their attachment mechanisms. One of our goals therefore is to provide mechanistic justification for a network generation mechanism that is easily implementable with local knowledge, and that can explain several of the social network features considered important in prior work, including short mean path length [16], heavy-tailed degree distributions [17], and high local clustering [19].

Triadic closure, i.e. forming a new link to a neighbor of a neighbor on the network, is an agent based, local mechanism common in realistic social settings [23, 25, 45]. It is known that “trust” in the resulting relationship and the associated “social capital” form a rationale for the appearance of triadic closure in social contexts (among opportunity and incentive for removing conflict) [18, 46], although the diffusion of information as a function of network structure has not been modeled explicitly in this context (*p* is taken as 1). Generative network models based on triadic closure with only few additional parameters can reproduce the characteristics of real social networks quantitatively [23–25, 47–50]. In this work, we do not aim to provide a more realistic algorithm for network growth, or one that more closely reproduces or fits real networks than existing work. Instead, we motivate TBA as a network growth mechanism based on indirect reciprocity, and then show that it is well approximated by triadic closure, which is easily implementable by agents with only local knowledge, and well studied in the literature as a realistic mechanism for network growth. Several properties of real social networks and their growth are not captured, including for example effects of homophily, node heterogeneity, or spatial constraints [51–54].

We provide further review of related work and the relationship to this paper in S1 File. The rest of the main text is organized as follows: we first describe and study our proposed model of reputational information spread. Later, we use the results to motivate trust-based attachment. Finally, we study the networks generated by TBA and show that triadic closure forms a simple and practical heuristic for TBA.

## Model of reputation spread

**Fig 1:**
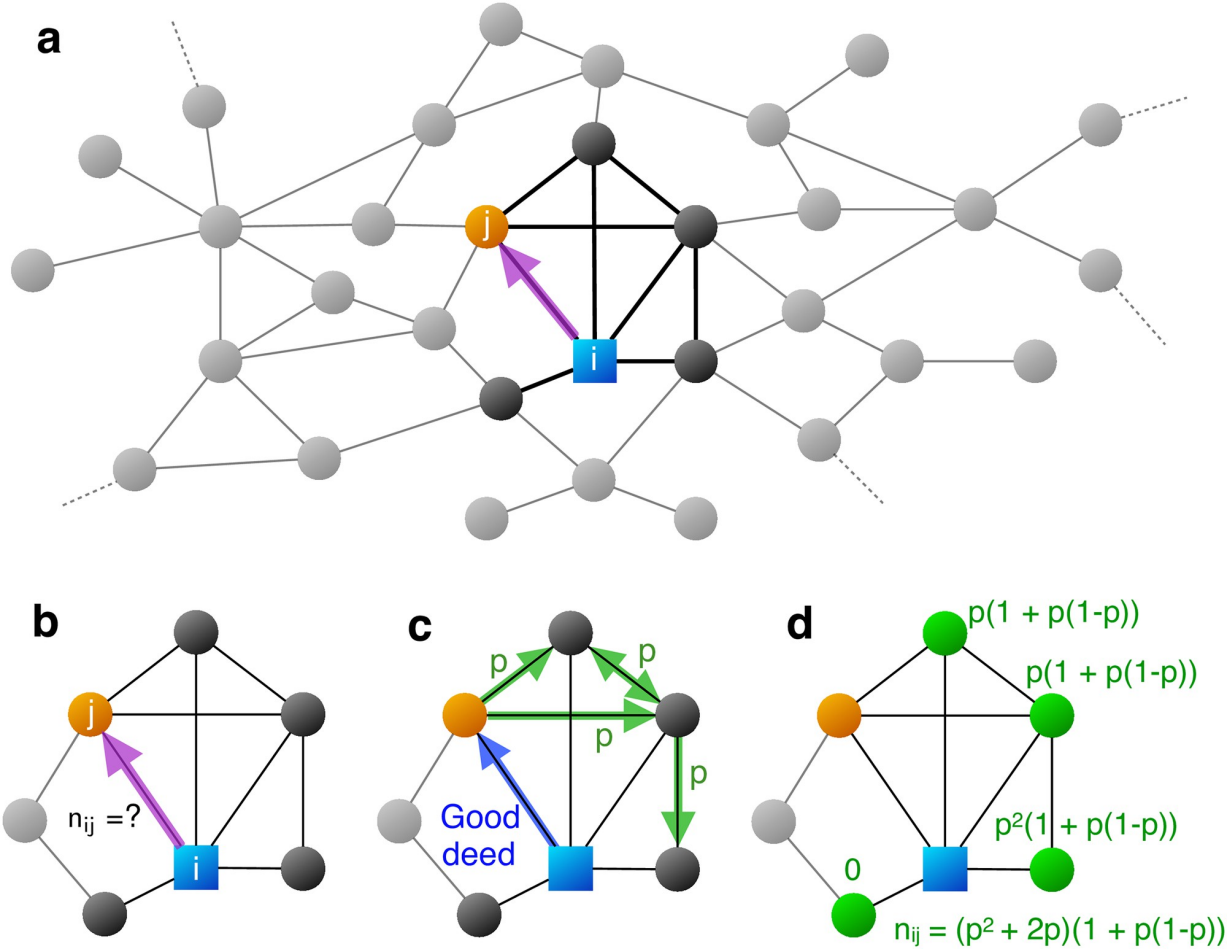
Calculating the size of the audience of a good deed. **a,** Individual *i* (blue square) is connected to individual *j* (orange circle) on a social network. **b,** If *i* performs a cooperative act toward *j*, who among the neighborhood of *i* (dark nodes and edges) will find out about it? **c,** We assume that gossip originates from the recipient, *j*, and flows with probability *p* to other individuals as long as they know the donor, *i*.**d,** Depending on the structure of the network, individuals have certain probabilities to learn about the cooperative act (shown in green). Summing over these probabilities gives the expected number of individuals *n*_*ij*_, which is the size of the audience for an action from *i* to *j*.

Consider a population of individuals occupying the nodes of a graph. The edges determine mutual acquaintance, which implies possibilities of social interaction and communication [55]. On the background of this social structure we consider a game of gossip and social information spread (Fig 1). An individual, the “actor”, has the option to perform a cooperative act for one of her neighbors, the “recipient”. Other individuals learn about the good deed in the following way: the gossip originates from the recipient J(we do not consider here the effect of the originator directly advertising their own action); each individual, who knows about the good deed, transmits the information to each of its neighbors with probability *p*. We assume that information can only flow between two individuals who both know the actor [14]. In S1 File section “Global spread” we study the consequences of relaxing this constraint: as the probability of gossip spreading beyond neighbors of *i* increases from 0, global structural properties of the network become increasingly important.

For a given graph, our model has a single parameter, *p*, the probability of gossip transfer over an edge. The basic calculation that we perform is the following: for a given donor *i* and recipient *j* on the graph, what is the expected number, *n*_*ij*_, of third party individuals that learn about the cooperative act Jwith information originating from the recipient *j*. The quantity *n*_*ij*_ is the expected size of the Jreputational audience of *i*’s action toward *j*. The larger the value of *n*_*ij*_ the more incentive there is—based on considerations of reputation and indirect reciprocity [1, 6]—for a cooperative act from *i* to *j*, both due to the threat of punishment for uncooperative behavior and the possibility of reward for cooperative behavior. In interpreting *n*_*ij*_, we explicitly assume that there will be sufficient upcoming future interactions to make these considerations of the future relevant/dominant.

We have

$$\begin{array}{c} n_{ij}=\sum_{k\neq j}P_{jk} \end{array}$$ (1)

The index *k* runs over all neighbors of the individual *i*, but omitting *j*. The quantity *P*_*jk*_ denotes the probability that gossip [14] originating from *j* reaches *k*. *P*_*jk*_ grows with the number of paths that can carry information, but falls off with their length. Note that *P*_*jk*_ is the percolation probability with parameter *p* between node *j* and *k* on the sub-graph that is given by neighborhood of individual *i*. Calculating percolation on general graphs is a well-known, formidable problem, and efficient algorithms exist only for special cases [14, 56–58]. However, in S1 File section “Random neighborhood approximation for gossip propagation” we derive a fast and accurate approximation for calculating *n*_*ij*_ as a function of the local degree, clustering, and embeddedness by leveraging the reliability theory of random graphs [56]. We test the applicability of this approximation by comparing it to exact calculations on small graphs and to extensive numerical simulations of both real-world and artificial networks (Extended Data Figs 1 and 2 in S1 File).

**Fig 2:**
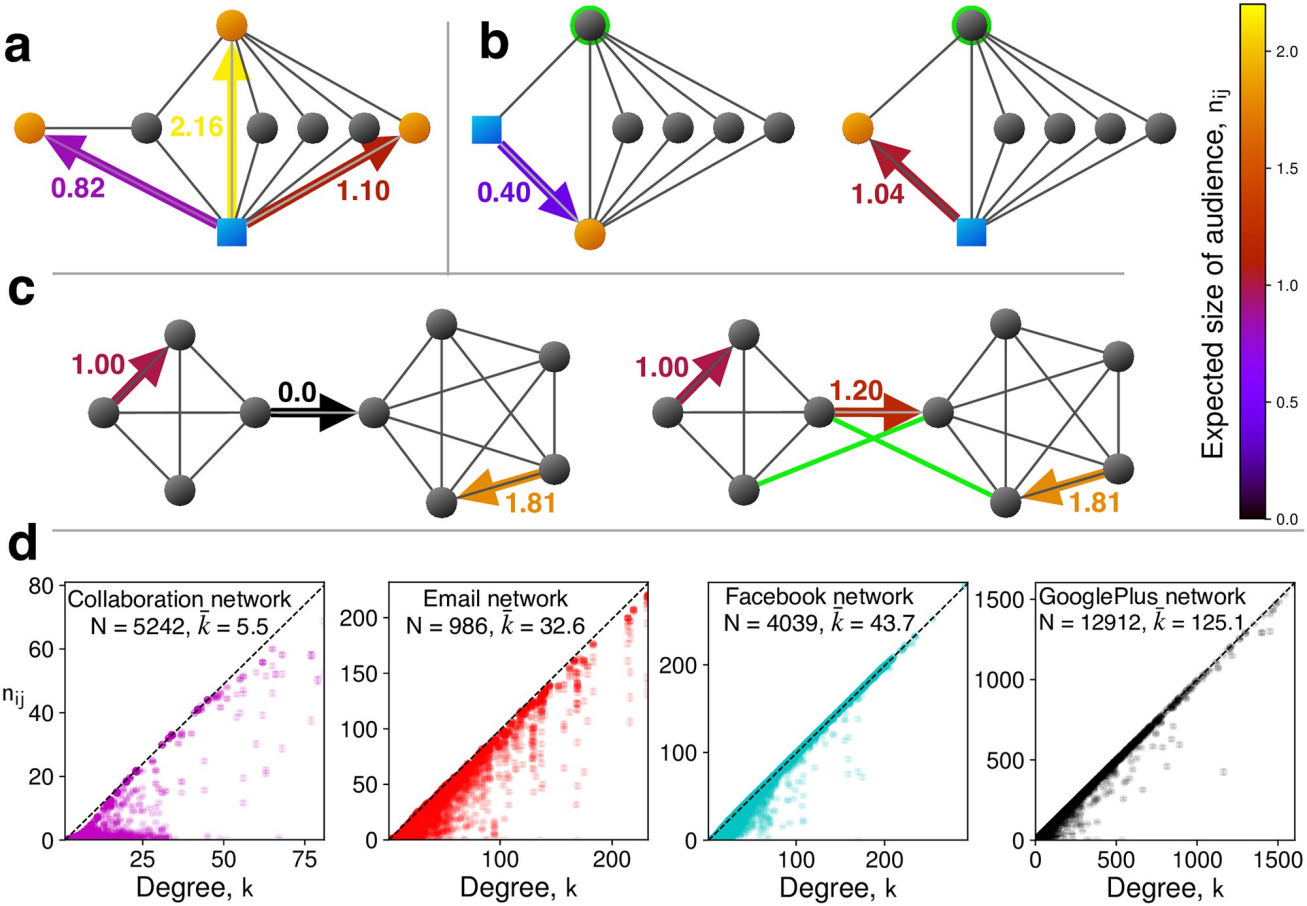
Properties of *n*_*ij*_ on local structures and real-world networks. Values of *n*_*ij*_ are shown as the colors of the respective arrows. **a,** the value of *n*_*ij*_ varies between recipients (orange circles) of the same actor (blue square). The coloring is as in Fig 1 description. If the recipient, *j*, is central to the neighborhood of the actor, *i*, and connected to several others, then *n*_*ij*_ is large (yellow arrow). For a recipient, who is only peripherally connected, *n*_*ij*_ is lower (red arrow). **b,** Jthe value of *n*_*ij*_ can differ from *n*_*ji*_. In both cases, the overall networks are identical and the actor and recipient have a single mutual neighbor in common (highlighted in green). In the left case, the mutual neighbor is the actor’s only additional neighbor, and *n*_*ij*_ is low. In the right case, the actor has several other neighbors who can learn about the interaction, and *n*_*ij*_ is higher. **c,** the value of *n*_*ij*_ is large between nodes that are part of the same highly connected community (magenta and orange arrows). Two such communities may only be weakly connected to each other, and thus *n*_*ij*_ is low for inter-community interactions. By adding some new links (thick green edges), higher incentives to cooperate (red arrow) can be established. Enhancing the interconnectivity between communities builds trust. Parameters: *p* = 0.4. For clarity we here omit coloring actors and recipients separately. **d,** Values for *n*_*ij*_ are shown for 1000 random edges on various real social networks [59, 60, 64] (see S1 File for details). Highly connected nodes have values of *n*_*ij*_ near the maximum possible *k* − 1 (see “Size of the expected audience” in S1 File; black dashed diagonal). The total number of nodes *N* and the mean degree $\bar{k}$ are given for each network. Parameters: *p* = 0.2.

In Fig 2, we show various examples of network structures and make the following observations. (i) For a given actor, *n*_*ij*_ can vary strongly between recipients. Links to individuals who are weakly connected with or even isolated from the rest of the actor’s neighbors receive only small values of *n*_*ij*_. By contrast, links to neighbors that are centrally located and highly connected to many other neighbors carry large values of *n*_*ij*_. Such individuals can distribute information widely and thereby generate large effective audience sizes. (ii) The embeddedness [16, 18] of edge *ij*, which is the number of mutual neighbors between nodes *i* and *j*, has has a strong predictive influence on the value of *n*_*ij*_ (see Extended Data Figs 1 and 2 as well as “Size of the expected audience” in S1 File). (iii) The value *n*_*ij*_ can be very different from *n*_*ji*_. Some links can have good incentives for cooperation in one direction but not in the other. (iv) The value *n*_*ij*_ can be changed dramatically by modifying just a few key connections. (v) Overall, having actor and recipient be part of a large, densely interconnected community results in the largest values of *n*_*ij*_.

In Fig 2, we also illustrate the behavior of *n*_*ij*_ on four real-world social networks. These networks represent professional (“Collaboration” and “Email”) [59] and personal (“Facebook” and “Google Plus”) [60] relationships comprising thousands of individuals, each of which with tens to hundreds of links. We find that well connected nodes universally have high values of *n*_*ij*_ towards their neighbors. On real social networks, gossip almost surely percolates throughout sufficiently large neighborhoods [14].

For every directed edge on a social network, the parameter *n*_*ij*_ is the expected size of the audience who find out about a cooperative act from *i* to *j*. Thus, *n*_*ij*_ determines the incentive for individual *i* to be cooperative toward individual *j* based on reputational consequences. Conversely, *n*_*ij*_ also determines the trust of *j* that *i* will cooperate [61]. While various notions of “trust” have been defined [62], here we use the word in the particular sense that we expect that another player will be cooperative.

In S1 File, we also extend our model to the case of “global cooperation”, where an individual has the option to perform a cooperative act such as a public service or a donation that is not directed towards any particular recipient. In that case, the act may be observed by any of the individuals’ neighbors and gossip can then originate (see the section “Global cooperation” and Extended Data Figs 3 to 5 in S1 File).

## Trust-based attachment

**Fig 3:**
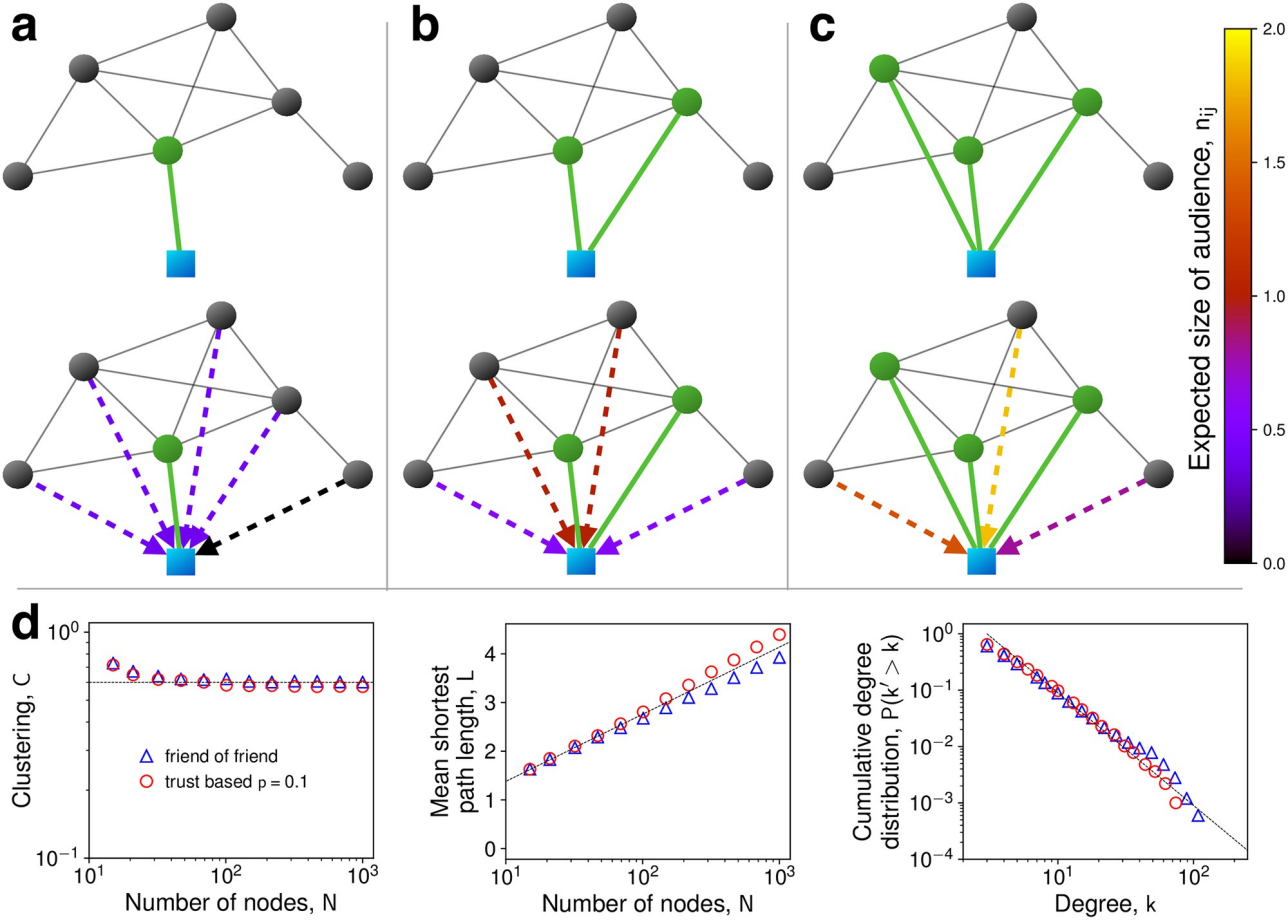
Trust based attachment. The attachment process **(a-c)** begins by a new individual (blue square) being introduced to a social group by a random individual (green) of that group. The new individual *j* selects additional friends *k* proportional to the trustworthiness of that link—that is the value *n*_*kj*_—if it were formed. The broken lines, in color, indicate the values of *n*_*kj*_ on the edges that could be formed. Every time the new individual actually forms a link, the values of *n*_*kj*_ for future potential friends change. The new individual in this case selects two additional friends for a total of three connections (green). **d,** comparing graphs generated by TBA (red circles) and by friend-of-friend attachment (blue triangles). The TBA graphs display high and constant (as *N* → ∞) clustering. The dashed line indicates the limiting value. The networks also show small world behavior, i.e. logarithmically growing mean shortest path length (the dashed line indicates *logN* behavior), as well as a power law degree distribution (the dashed line has a slope of −2). Trust based attachment generates some of the same structural features as exhibited by real-world social networks, even in the simplified local form of friend-of-friend attachment. Error bars in both plots are significantly smaller than the size of the symbols. Parameters: *p* = 0.1, *k* = 6, *N* ∈ [15, 2000]. For other values of *p*, see Extended Data Fig 6 in S1 File.

We now use these insights to propose a mechanism for generating social networks (Fig 3). Consider a newcomer who is introduced to a social group by a random individual from that group. Thus, the newcomer’s first link is formed to a random individual. Subsequently, the newcomer seeks to attach to individuals who have a high incentive to be cooperative toward her [18, 46, 63]. This goal can be achieved in the following way. The newcomer, *j*, attaches to individual *i* with a probability that is proportional to the quantity *n*_*ij*_ on the potential link. The larger *n*_*ij*_ the more incentive for *i* to cooperate with *j* and thus the more reason for *j* to trust that *i* is cooperative toward her. We assume here that the target individual for the new attachment *i* only accepts newcomers with a given constant probability. As this does not influence the attachment statistics (but only the effective time scale of the process), we omit this influence in the following discussion.

In order to generate a network of *N* total individuals with average degree *k*, we begin with a fully connected graph of *k* + 1 individuals. Then we add individuals one at a time until there are *N* individuals in total. Each new individual forms *k*/2 connections as described above. We propose to call this algorithm for generating social networks “trust based attachment” (TBA). In this algorithm, trust forms the glue of social networks.

Naturally, TBA forms a simplified model that does not capture all aspects underlying the formation of real-life social networks. Therefore, we do not expect this model to reproduce all features of real social networks. For example, it does not reproduce the distinct community structures commonly found in real social networks, possibly due to a lack of modeling homophily, spatial constraints, or heterogeneity in the properties of individuals [51–54]. It is also not an attempt to quantitatively “fit” the properties of real social networks. Instead, we aim to show that this simple mechanism generates several important properties of real social networks, thus providing a possible mechanistic justification for their emergence. In particular, TBA generates networks that have several desirable, realistic features (Fig 3 and Extended Data Fig 6 in S1 File), which include include high clustering, power law degree distributions, and small world behavior.

**Fig 4:**
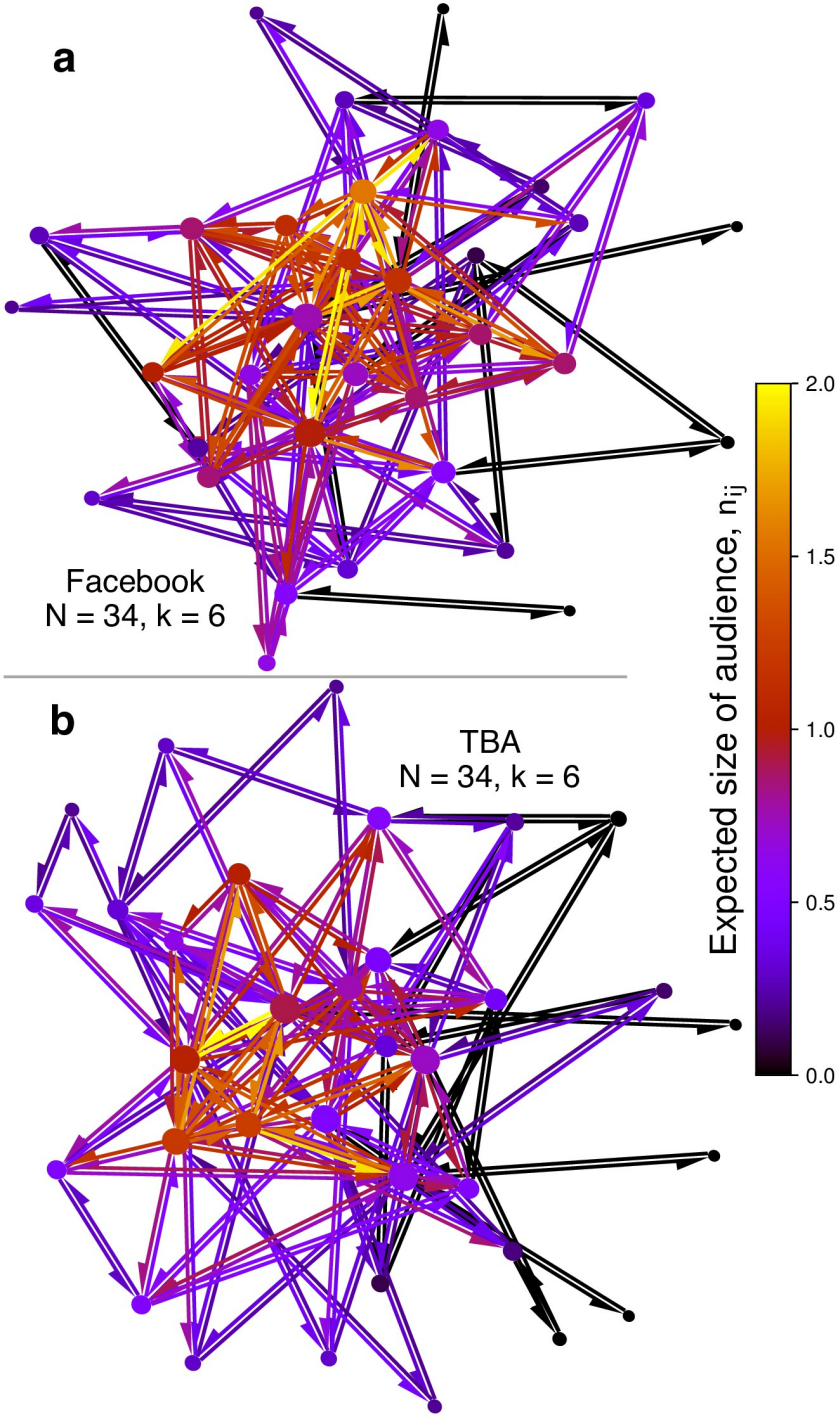
Illustrating *n*_*ij*_ on a Facebook and a TBA network. A subset of a Facebook social network [60, 64] is shown at the top, and a network generated by TBA with the same number of nodes (*N*) and average degree (*k*) is shown below. Edges are colored by *n*_*ij*_ and nodes are colored by the value of *n*_*ij*_ averaged over all neighbors *j*, which we denote as *n*_i*_. While *n*_*ij*_ is specific to the relationship from *i* to *j*, *n*_i*_ can be seen as a measure of “average trustworthiness” of the individual *i*. JWhile these networks are quite small, they allow a clearer view into the local structure. The networks have strongly connected central hubs and high clustering. The highest values of *n*_*ij*_ appear with strongly embedded nodes in the center, while the lowest values appear with the isolated peripheral nodes. Parameters: *p* = 0.2. See Extended Data Figs 7 and 8 in S1 File for more details.

To build some further intuition for the key quantities and network models introduced in this paper, we show in Fig 4a real examples a component of a real social network as well as an equivalent network of the same size and average degree generated by TBA. The purpose of this illustration is not to show quantitative agreement. Rather, it is to illustrate the qualitative structure of these networks, and show the dependence of *n*_*ij*_ on local structural features, embedded in a more realistic context than in previous figures. Edges are colored by *n*_*ij*_ and nodes are colored by the value of *n*_*ij*_ averaged over all neighbors *j*, which we denote as *n*_i*_. While *n*_*ij*_ is specific to the relationship from *i* to *j*, *n*_i*_ can be seen as a measure of “average trustworthiness” of the individual *i*, and its statistics have been studied extensively on real-world as well as small world and preferential attachment networks [14]. Even in the small networks in this figure one can see the varied degree distribution and the densely connected and highly clustered “core”. For a more quantitative assessment of networks generated by TBA we refer to Fig 3.

TBA captures Ja subset of the qualitative features of real social networks well, including high clustering, small world behavior, hubs, varied degree distributions and an overall high prevalence of trust [16–21, 50]. Nodes on the periphery of the social networks with only few attachments to distinct parts of the network have low values of *n*_i*_. By contrast, highly connected nodes that are part of densely clustered communities display large trustworthiness.

## Triadic closure as a plausible heuristic for TBA

Implementing TBA in practice would require individuals to have extensive knowledge of the local network structure and to perform complicated calculations. However, we find (see Fig 3, as well as Extended Data Fig 6 and “Network generation” in S1 File) that the simple heuristic of attaching to a random neighbor of a random neighbor (“triadic closure”) can accurately approximate TBA for values of *p* ≲ 0.2. This “friend-of-friend” attachment is simple and intuitive, and can be implemented by individual agents without global knowledge of the network structure [35]. Both methods generate networks with very similar connectivity statistics, including high clustering, short mean path length, and power law degree distributions (see Fig 3). For triadic closure, the high clustering property emerges since it forms edges that are biased to be highly embedded, which means forming many triangles at once. The random shortening of paths with every additional edge creates the small world behavior. Attaching to a random neighbor of a node already involved in the network introduces a bias towards high degree nodes [18], which reproduces the preferential attachment bias and thus power law degree distributions. For large values of *p* > 0.2, we find that TBA still generates networks with nearly the same properties as friend-of-friend attachment, except that the degree distribution for TBA departs from a power law and includes even more high-degree “hubs” (see “Network generation” and Extended Data Fig 6 in S1 File).

In summary, the incentives created by indirect reciprocity suggest triadic closure as a simple and practical heuristic for creating links with high trust. Triadic closure in turn can explain multiple characterstics of real social networks [23–25, 45, 47–50]. The key structural features connecting the spread of indirect reciprocity to triadic closure are related to the quantity *n*_*ij*_, which in turn can be well approximated with knowledge of the local degree, clustering, and embeddedness for a given node.

In principle, there are many ways to build social ties: individuals could strive to form long range, random connections to increase diversity; seek out individuals of a certain type, status or connectivity; or look for meeting venues that bring together people with specific interests or motivations. While all these effects may play a role, our work helps explain why out of all the ways that humans could create new social connections, forming relationships with friends of friends is a natural, intuitive, and trust-inducing process, and one that is likely to generate trustworthy and cooperative connections.

## Supporting information

S1 File This file contains supplemental discussions including sections with additional information on (i) the relationship with past work; (ii) details on methods and algorithms used; (ii) the calculation of the size of the expected audience; (iii) model assumptions and extensions; (iv) quantitative derivations around network generation; as well as (v) extended data figures. (PDF)
