# Supplementary material for: Trust based attachment
Source: PLoS One. 2023 Aug 23;18(8):e0288142. doi: 10.1371/journal.pone.0288142 (PMC10446209; doi:10.1371/journal.pone.0288142)
Supplement: S1 File — (PDF) [file pone.0288142.s001.pdf]

Trust based attachment

Julian Kates-Harbeck[1], Martin Nowak[2,3*],

1 Department of Physics,
2 Department of Mathematics,
3 Department of Organismic and Evolutionary Biology, Harvard University, Cambridge MA 02138, USA

* martin_nowak@harvard.edu

Supporting information

SI Guide

Supplemental Information This file contains supplemental discussions including sections with additional information on (i) the relationship with past work; (ii) details on methods and algorithms used; (ii) the calculation of the size of the expected audience; (iii) model assumptions and extensions; (iv) quantitative derivations around network generation; as well as (v) extended data figures.

Relationship with past work

Our paper studies the relationship between indirect reciprocity and network structure. In the first part, we study how information spreads across an individual's neighborhood in order to quantify the expected size of the audience of a given interaction. In the second part, we use these insights to study trust based attachment.

Past work on indirect reciprocity

The standard approach to indirect reciprocity assumes well mixed populations [1–12] and does not take the effect of network structure on information flow into account.

Past work on information flow on networks

Many papers have studied information flow and the spread of contagion on networks [13–19], but these studies do not relate the spread of information to gossip and indirect reciprocity. Moreover, we study specifically the information spread as restricted to the neighborhood of any one given node. The work of Lind *et al.* [20] does study this form of information spread. They consider the following model. For a given individual i who is "victim" of the gossip, a neighbor j spreads malicious gossip to all mutual neighbors of i and j. In the next round, all those individuals spread the information to their mutual neighbors with the victim i, and so on. They average this process over all possible originators j from i's neighborhood. They then study the fraction of neighbors of i that are eventually reached by the gossip (f), as well as the mean number of rounds to reach them (τ), as a function of the degree of node i on various real and artificial networks [21]. They find that for several network types, there exists an ideal degree for i such that the spread of malicious gossip (as quantified by f) is minimized. They present various modifications of their original model in later

sections. There exists an interesting relationship between their f in the updated model introduced in the last 2 paragraphs of section IV, and our quantity n_{ij}:

$$f = \frac{1}{k_i} \left(1 + \sum_{j \in \mathcal{N}(i)} n_{ij} \right) ,$$

where k_i is the degree of node i and $\mathcal{N}(i)$ is the set of neighbors of node i. The authors do not relate their analysis of gossip spread to indirect reciprocity and also do not consider the effect of gossip on network growth and attachment. By contrast, these are key components of our paper. Moreover, we consider information propagation for each neighbor of each focal individual (i.e. for all edges) on the network and analyze the local structural features affecting the spread.

Shaw *et al.* [22] study analytically and numerically the effect of random gossip on network structure, due to the "bonding" (i.e. the strengthening of ties) that occurs between two individuals who gossip about a third party "victim", and the weakening of ties between the victim and the gossipers. They find that if gossip spreads far, it helps build more cohesive clusters and strengthens triads, while it otherwise destroys triads. This influence of "bonding" is distinct from that of indirect reciprocity and thus forms a complimentary direction of study.

Past work on indirect reciprocity on networks

Several papers study models including information flow and link formation on networks in the context of repeated games [23–26]. The authors focus on how an individual may update links based on the past behavior of other agents in the repeated game, and how the resulting networks co-evolve with individuals' strategies. Their finding that more cohesive, tightly clustered networks go hand in hand with cooperative behavior [24, 25] is in line with our result that such structures lead to higher values of n_{ij}. These papers do not study directly which network properties enhance or suppress the spread of gossip.

Novelty of our approach

- We model the spread of gossip on social networks in the context of indirect reciprocity. This leads to an expected audience size n_{ij} that is specific for any interaction between any pair of individuals i and j on the network.
- Our paper asks how network structure influences the power of indirect reciprocity. We explore the behavior of n_{ij} for individual edges, and describe its dependence on local structural features of the neighborhood.
- We derive analytical approximations for the expected audience size n_{ij} as a function of local network parameters.
- Due to indirect reciprocity, we consider the expected audience size as a measure of the incentives (the "threat" of reputation consequences or the temptation of reputational rewards) influencing an actor's future behavior towards a recipient.
- Our approach models the effect of indirect reciprocity on the growth dynamics of social networks. We assume that individuals seek attachments where their neighbors have a high incentive to be cooperative due to the ability of the local network to conduct gossip. This results in the network growth mechanism of trust based attachment.

- In contrast to updating existing links given past experience, TBA represents a strategy for forming the first links in a novel network setting, thus complimenting the above body of work.
- Triadic closure is shown to be a good heuristic approximation for TBA.

Past work on models of network growth

There is an extensive literature on models of network growth that can reproduce various aspects of the statistics [27] of real world networks. Small world behavior can arise with a small number of random long-range connections in otherwise densely locally connected networks [28]. Models based on randomly connected groups of communities of various sizes and densities [29] can reproduce both clustering and degree statistics of target networks, although they require a global (i.e. not agent-based) algorithm to generate networks. Agent based models have shown scale free degree distributions to emerge from preferential attachment [30]. A trade-off of popularity and similarity can accurately model additional properties of networks such as their hyperbolic geometry and high local clustering [31]. These models however require global knowledge of the network (such as the degree of all nodes) for an agent to implement their attachment mechanisms. Triadic closure, i.e. forming a new link to a neighbor of a neighbor on the network, is an agent based mechanism common in realistic social settings [32–34]. Generative network models based on triadic closure with only few additional parameters can reproduce the characteristics of real social networks quantitatively [34–38].

Novelty of our approach

- Our key innovation is to use indirect reciprocity to motivate growth.
- Our approach motivates trust based attachment by considering individuals' incentives in the presence of indirect reciprocity and reputation spread. Unlike all the above approaches, this provides a game theoretic justification for TBA as an attachment strategy.
- Our approach directly motivates the well known method of triadic closure (friend-of-friend attachment) by showing that it is a good heuristic approximation to TBA.
- Friend-of-friend attachment is a local, agent-based attachment algorithm which does not require global knowledge, whose only parameters are the total size of the network N and its average degree k, and which is fast and easy to implement with a runtime complexity of only $O(Nk)$.

Numerous authors have studied indirect reciprocity, the spread of gossip and information on social networks, as well as models of network growth and attachment. Here we present an analysis that joins indirect reciprocity and network growth and thus provides a missing link between those literatures.

Methods and Algorithms

Calculating n_{ij}

In general, computing n_{ij} has exponential running time in the number of edges of the actor's neighborhood. We employ two methods for calculating n_{ij} in this paper. For

sufficiently small local neighborhoods (if the degree of an actor node is $\lesssim 8$), we calculate n_{ij} exactly by enumerating all possible combinations of which edge is sampled in the neighborhood graph, calculating the audience size in that case, and summing all those values weighted by the probability of sampling those edges. For larger neighborhoods, we use Monte Carlo sampling to evaluate n_{ij}. For N_{trials} repetitions, we sample the neighborhood graph (each edge is kept with probability p) and count the size of the audience. The values are summed up and the mean value over all N_{trials} repetitions is reported. For all experiments we use $N_{trials} = 10^4$.

Testing the approximation for n_{ij}

We test numerically our assumptions and find that they hold well over a wide range of the relevant parameters, and all the different graph types that we tested. Using Monte-Carlo simulations, we find the exact value of P in the context of the different reputation propagation models defined above (mutual neighbors only, local neighborhood model). These values are computed for several nodes sampled randomly from various different graph types, including Erdos-Renyi random graphs, Watts-Strogatz small world networks [28], Random Gaussian Geometric Graphs (obtained by placing nodes uniformly at random in a square 2D region with periodic boundary conditions, and forming links with a Gaussian probability distribution as a function of Euclidean distance between node pairs, normalized such that the overall average degree on the network becomes some specified k), preferential attachment graphs [30], graphs generated by trust based attachment as described in this paper, and a subset of the social network graph from Facebook [39]. The results are shown in Extended Data Fig. 1. The agreement between simulations and theory is universal, for all nodes and edges on all graphs studied. Our analytical and numerical results imply that for any node on any social network, the simple parameter combination of k, c, and m_{ij}, gives a very strong and precise prediction of the expected audience size n_{ij}.

Generating social networks

Let us consider an individual in a population subject to an interaction structure. Given that the structure of social networks has a strong influence on other individuals' incentive to cooperate, how should our individual best attach themselves to other individuals? How can the individual exploit local population structure to increase their payoff [40, 41]? The individual wishes to chose their neighbors such that these neighbors have a high incentive to cooperate, since this maximizes the payoff for the individual in question. Recall that n_{ij} can be interpreted as a measure of the incentive of individual i to cooperate with individual j (note the directionality). Thus, it is a measure of *trust* between j and i. The higher this value, the more j can trust that i will cooperate (because it would suffer the reputational consequences otherwise). We consider the following specific algorithm inspired by this trust based attachment. A node j enters an existing population encoded as a network. It then attaches $k/2$ edges into the population, one after the other, where the neighbors for these edges are chosen as follows. For every node i on the graph to which j is not connected yet, n_{ij} is computed. One node is then sampled from these candidates proportional to its value of the trust measure n_{ij}, and an edge is formed to that new neighbor. The average clustering of the resulting graph can be controlled by the weighting of n_{ij}. The probability of picking a neighbor i can in principle be a *nonlinear* function of n_{ij} that emerges from the importance of certain connections, how likely we are to keep them, or how often introductions are made; [32] for example is a threshold model in n_{ij}. We find using numerical simulations that the limiting value of the clustering coefficient is

an increasing function of how strongly (i.e. compared to linear) the probability of attachment increases with n_{ij}.

For the first of the $k/2$ edges the node j won't have any neighbors yet and thus all n_{ij} will be zero. The first neighbor is therefore chosen at random. The results are not sensitive to this choice. The resulting networks show the same statistics if the first neighbor is chosen according to preferential attachment. To grow a population of N individuals, we begin with a fully connected graph of $k+1$ individuals (to ensure that the average degree of the resulting network is exactly k) and then add one node at a time with $k/2$ edges each as described above until we have N total individuals. The result of this attachment model are shown in **Figure 3**.

This simple trust based attachment model generates graphs that show key characteristics of social networks [35]: small world connectivity (i.e. path length that scales like $\sim logN$), high clustering coefficient that approaches a constant value as N grows, and power-law scale free degree distributions.

Computing the exact value of n_{ij} is computationally intensive and not necessarily a viable strategy for an individual with imperfect information about the surrounding social network. In the **SI** section "Size of the expected audience", we provide an approximate analytical expression based on n_{ij}, c_i and k_i. Moreover, we can find the following very simple approximation for n_{ij}:

$$n_{ij} \approx pm_{ij} \ .$$

This approximation is obtained in the limit where $p \to 0$ or where we only consider paths of gossip spread of length at most 1. Either limit thus leads to the very simple rule that the probability of node j picking a new edge to node i is proportional to m_{ij}, the embeddedness of the edge ij.

This rule can now be interpreted (and implemented) as a very simple strategy by an individual j. For every new edge that node j wants to add, do the following: 1) pick a random neighbor; 2) pick one of their random neighbors; 3) Attach to them if there isn't already an edge. Repeat until $k/2$ edges have been formed. We show below that this is equivalent to picking a neighbor proportional to m_{ij}. This is a very intuitive and easy to implement rule: simply attach yourself to a random friend of a friend. **Figure 3** demonstrates that both trust based attachment with its full complexity, as well as the approximation based on attaching to a friend of a friend, result in the same macroscopic network properties qualitatively and quantitatively: high clustering, power law degree distributions, and small average path length. Moreover, it is simpler than preferential attachment in that it does not require global information. A naive implementation of friend-of-friend attachment for generating a network of average degree k with N individuals has $O(Nk)$ runtime, compared with $O(N^2k)$ for preferential attachment. Thus, this algorithm can be used to quickly generate realistic social network-like structures.

Plotting social networks

The data for the social networks is taken from the Google Plus, Facebook [42], EU research email, and General Relativity collaboration networks [43] obtained from the Stanford Large Network Dataset Collection [39]. For the Google Plus network, we use the first 10^6 edges. For the other networks, we use all edges.

Since visualization of such large networks is not practical, we focus on small subgraphs of these large networks when generating plots. In particular, we use the following approach to subsample large networks to desired values of N and k for plotting purposes. We first divide the larger network into communities of various sizes by running a standard community detection algorithm [44], and then find the

community closest in N to the desired value. We then remove random edges until the mean degree is as close as possible to k, and keep the largest connected component as our final sub-sampled network. For TBA, we first construct networks with the correct N and twice the average degree as desired and then run the same sub-sampling procedure to obtain the desired N and k. This provides a more fair comparison since all generated networks are now subject to sub-sampling, which can have effects such as introducing nodes with very few neighbors. This procedure is only used when we plot large example social networks and networks generated by TBA in **Figure 4** as well as Extended Data Figs. 5, 7 and 8. This procedure does not affect our computation of network statistics and metrics such as those shown in **Figure 3** or Extended Data Figs. 1, 3 and 6.

Size of the expected audience

Consider the game as described in **Figure 1** and the main text. We are interested in how i's decision of whether to act altruistically depends on the P_{jk}, the probability that information reaches k, another neighbor of node i, from node j, as long as all nodes on the path from j to k are connected to node i. Information is said to successfully travel between two nodes if there is a path of edges between those two nodes, where each edge independently and at random transmits information with probability p. Thus, P_{jk} is the edge percolation probability [20, 45, 46] with parameter p between node j and a given neighbor $k \neq j$ of node i, on the graph defined by $\mathcal{N}(i)$, the subgraph defined by the neighborhood of node i. Intuitively, if the P_{jk} are high, then neighbors are likely to find out about the good deed and reward the behavior. Thus i will have a high incentive to perform the act. On the other hand, if the P_{jk} are low, i will not have a large incentive to perform the good deed. The sum of P_{jk} over all neighbors measures the total expected audience size of a given action of node i:

$$n_{ij} = \sum_{k \in \mathcal{N}(i), k \neq j} P_{jk} \ ,$$

where $\mathcal{N}(i)$ denotes the neighborhood of node i. It can also be viewed as how much neighbor j can "trust" node i to be cooperative. It depends crucially on the network structure and the resulting ability of the network to diffuse reputation in the neighborhood of that or edge. Moreover, more effective information transmission (higher p) also makes altruistic action more favorable. Reputation systems have recently been scaled up to provide security and trust for transactions in vast and otherwise anonymous online communities such as Ebay, AirBnb, or Tripadvisor [47].

Random neighborhood approximation for gossip propagation

Suppose we have an actor node i, its neighborhood $\mathcal{N}(i)$, and a given recipient $j \in \mathcal{N}(i)$. We are interested in finding the edge percolation probability P_{jl} from j to a given other neighbor $l \in \mathcal{N}(i)$, $l \neq j$ on the subgraph defined by $\mathcal{N}(i)$. We assume that the following quantities are known: the degree k_i of node i, the local clustering coefficient c_i of node i (defined as the probability that two randomly chosen neighbors of i are connected to each other), as well as the embeddedness [28, 48] m_{ij} of the edge ij, which is the number of mutual neighbors of nodes i and j.

By definition, the size of the neighborhood subgraph is k_i and the number of edges on that subgraph is $c_i * \frac{k_i(k_i-1)}{2}$. Moreover, the number of edges between the recipient (the originator of the percolation) and the rest of the neighborhood subgraph is by definition n_{ij}.

We now make the following key simplifying assumption: We assume that the edges are distributed randomly on the neighborhood subgraph (Extended Data Fig. 9). In particular, we imagine that the recipient j is connected to each of the rest of the $k_i - 1$ neighborhood nodes with a probability of $\frac{m_{ij}}{k_i-1}$. This results in m_{ij} edges in expectation, which is the true number of such connections. Moreover, we assume that the rest of the $k_i - 1$ nodes are connected randomly (like an Erdos-Renyi random graph) among each other with probability

$$c^* = \frac{c_i \frac{k_i(k_i-1)}{2} - m_{ij}}{\frac{(k_i-1)(k_i-2)}{2}} \ .$$

This again results in the true number of remaining edges $c_i \frac{k_i(k_i-1)}{2} - m_{ij}$ in expectation.

In the percolation process, each of these edges is now kept with probability p. Thus, in our approximation, the final subgraph after edges have been sampled for the percolation process now has random edges from the recipient to the rest of the neighborhood with probability

$$p_1 \equiv p \frac{m_{ij}}{k_i - 1} \ ,$$

and edges among the rest of the neighborhood with probability

$$p_2 \equiv p \frac{c_i \frac{k_i(k_i-1)}{2} - m_{ij}}{\frac{(k_i-1)(k_i-2)}{2}} \ .$$

Without knowledge of m_{ij}, the equivalent best estimate would be to assume $p_1 = p_2 = cp$.

While these quantities are on average correct, the “random graph” assumption specifically assumes that there is no significant connectivity structure in this neighborhood (such as disconnected communities, etc.). Our assumption gets worse the less random the structure of the neighborhood is. This can be measured for example by the mean clustering coefficient on g. If it is that of a random graph (the lowest possible value), percolation is the most likely and our assumption is most accurate. The higher the clustering, the more our assumption overestimates P. Moreover, our assumption overestimates the percolation probability if the edges m_{ij} are concentrated among a separate, tightly connected subset of the neighborhood instead of being randomly distributed among the whole neighborhood.

The final expression for the percolation probability is

$$P_{jl} = 1 - \sum_{k'=1}^{k_i-1} A_{k'}(p_2) \binom{k_i - 1}{k'} (1 - p_2)^{k'(k_i-1-k')} \frac{k'}{k_i - 1} (1 - p_1)^{k'} \qquad (1)$$

where $A_j(x)$ is the reliability function [49] that a graph of size j is fully connected given that all edges exist independently and at random with probability x. This approximation of P_{jl} is the same for all l. The final expectation value n_{ij} is a sum over all $k_i - 1$ nodes on g:

$$n_{ij} = (k_i - 1) P_{jl}$$

with P_{jl} as given above.

We now derive Eq. (1). The recipient is connected to the each node in the rest of the neighborhood with probability p_1. The other neighbors among each other are connected with probability p_2. We thus have a situation with a random graph of $k_i - 1$ nodes, connected among each other with probability p_2 — let us call this graph g — and a single outlying node (the recipient) connected to every node in g with

probability p_1. P_{jl} is now the probability whether there is any path from the outlying node to a randomly chosen one of its neighbors. This situation is illustrated in Extended Data Fig. 9.

Let us find the complement probability – that a randomly chosen node — call it l — on g is not connected to the recipient (i.e. there is no path between them). In particular, consider a connected subgraph of g of size exactly k'. We will find the probability that node l is in this subset of size exactly k', and that the recipient is *not* connected to it.

Pick a subset of k' nodes. The probability of this subset being connected is $A_{k'}(p_2)$, and there are $\binom{k_i-1}{k'}$ such subsets. The probability that this subset is disconnected from all other $k_i - 1 - k'$ nodes on g is $(1-p_2)^{k'(k_i-1-k')}$ (i.e. the connected subset has size exactly k'). With probability $\frac{k'}{k_i-1}$, the node l is part of this subset. We need to make sure that the recipient is disconnected from all nodes in the subset – this is given by probabilty $(1-p_1)^{k'}$. Thus, a single term (for a given k') in the sum in equation 1 is the probability that node l lies in a connected subset of size exactly k', and that the recipient is not connected to this subset. By summing over all possible sizes k', we have listed all disjoint ways in which the recipient can be disconnected from node l. Thus, this is by definition $1 - P_{jl}$, giving equation 1.

In the case where $p_1 = p_2 = cp$, this reduces to the "two-terminal reliability function" $T_{k_i-1}(c_i p)$ [49], which measures the probability that two randomly chosen nodes on a random graph of size $k_i - 1$ with edge probability $c_i p$ are connected.

Limiting cases of P_{ij}

Consider the limit as $p \to 0$. In this limit, only paths of length 1 will contribute to the flow of information, since all other paths only exist with probability $O(p^2)$. By definition, there are m_{ij} edges (paths of length 1) from the recipient to the rest of the neighborhood $\mathcal{N}(i)$ of the actor. Each such edge independently carries information with probability p. Thus we have

$$n_{ij} = pm_{ij} + O(p^2) \ .$$

This expression becomes exact as $p \to 0$ and makes the strong dependence of n_{ij} on the embeddedness m_{ij} explicit. The dependence of n_{ij} on features of the neighborhood structure beyond the embeddedness is thus a "higher order" effect and becomes more important for larger p.

Model assumptions and extensions

As in any model, our approach involves several simplifying assumptions which we discuss here. Overall however our goal is not to model the full complexities of indirect reciprocity, but rather to isolate the impact of social network structure in particular on reputation diffusion and the consequences thereof on cooperation.

Global spread

We have assumed that reputation can only travel across nodes that are neighbors of the central node. One could also consider a model where reputation can travel across any path on the entire network. We consider a probability p_1 of transmitting gossip on the local neighborhood of the actor, and a probability p_2 on any other edge of the graph. This model reduces to our original "local" model with $p_1 = p$ as $p_2 \to 0$. This kind of global information spread would entail that people gossip about third parties

that they don't actually know (of). One example of this is two people talking about a celebrity.

In this case, the global structure of the network becomes relevant. An interesting tradeoff arises: there is now an exponentially growing number of possible paths for the reputation to flow, however, longer paths are exponentially less likely to exist. We thus have the percolation probability on the overall graph between two nodes becoming relevant. We find numerically that in this model the overall behavior of the percolation probability now depends on global parameters of the graph such as the overall number of nodes, mean degree and global clustering. A way to approximate the results would be to use insights from the global percolation properties of clustered networks [50].

We study this model numerically in Extended Data Fig. 10. We show example pairs of actors and recipients from various different networks, and the resulting values of n_{ij} with a given value of p_1 and as a function of p_2. As expected, the global percolation model reduces to the known local solution as $p_2 \to 0$. For finite values, the value of n_{ij} increases. The probability of information traveling from the recipient to another neighbor of the actor via edges outside of the actor's neighborhood depends on the percolation properties of the overall network.

We show the global edge percolation threshold p_c on the overall network (determined numerically), defined as the probability of keeping a given edge on the overall network for which the probability that two randomly chosen nodes remain connected via some path is 0.5. The connection probability of two randomly chosen nodes rapidly becomes 0 below this threshold, and 1 above this threshold. The transition becomes sharper as networks increase in size [45]. The actor is by definition a mutual connection between the recipient and any other neighbor of the actor. This introduces a bias that distinguishes the recipient and other neighbors of the actor from two random nodes. Nevertheless, we find that the boost in n_{ij} from the global percolation is strongly related to the global percolation probability as a function of p_2. In particular, we can in general distinguish the following regimes. For values of $p_2 < p_c$, the global value of n_{ij} is nearly the same as for the local case with $p_2 = 0$. For values of $p_2 > p_c$, n_{ij} rapidly approaches its maximum value $k - 1$. The only exception are cases where the network becomes disconnected once the actor node is removed, since the actor node and its edges are never counted in the transmission of gossip. In those cases, n_{ij} approaches some value $< (k - 1)$ for $p_2 > p_c$. Finally, there is an intermediate regime $p_2 \approx p_c$. Here the value of n_{ij} rapidly rises from its local value to the maximum possible value $k - 1$ with increasing p_2. The exact functional dependence depends on the full global network. Further aspects of possible models for the spread of gossip and their relation to global network properties are also explored in [20].

Overall, the question of who "knows of" another individual could also be tackled by considering directed networks in future work. Person A might know (of) person B but not vice versa, especially for the classic example of celebrities. Moreover the interaction graph might not be the same as the graph of who knows whom. Interactions and communication strength might be weighted. An individual with hundreds of connections may not be able to communicate with each neighbor to the same degree as an individual with only a few. While adding more parameters and complexity, a more complete model could thus consider directed and weighted graphs, as well as separate graphs for game interaction and reputation diffusion.

Global cooperation

In the main text, we assume that the good deed of the actor is directed towards a particular recipient individual, who is then the originator of gossip. We may also consider the case of global cooperation, where the good deed is not directed towards anyone in particular, such as helping a stranger in need, performing public service, or

making a donation. In this case, we consider the same model with the modification that every neighbor can independently and at random “observe” the good deed of the actor with probability q. In this case, all neighbors that observed the act serve as originators of gossip, which travels in the same way as in our original model with parameter p (there now of course can be several originators). For any given node i on the graph, the parameters q and p then define an expected audience size n_i for this global cooperation. This quantity measures the incentive for any individual on the graph to be globally cooperative.

If we again approximate the neighborhood of i as a random graph with k_i individuals and a connection probability of c_i, we can use similar reasoning as in the section “Random neighborhood approximation for gossip propagation” above to derive an approximate expression for n_i. In particular, let $\tilde{P}_i$ denote the percolation probability of the information reaching a randomly chosen neighbor j of individual i (the information needs to first travel to any neighbor of i via observation, and then possibly via gossip to the neighbor j). Then with the random neighborhood assumption we have the same exact situation as in the derivation of equation 1 above, with the replacements $p_1 \to q$, $p_2 \to cp$, and $k_i - 1 \to k_i$. We thus obtain

$$\tilde{P}_i = 1 - \sum_{k'=1}^{k_i} A_{k'}(cp) \binom{k_i}{k'} (1-cp)^{k'(k_i-k')} \frac{k'}{k_i} (1-q)^{k'} \ . \tag{2}$$

Since this is the probability of the information reaching any particular neighbor, the expected number of neighbors reached by the gossip is then simply

$$n_i = k_i \tilde{P}_i \ .$$

In Extended Data Fig. 3 we show that this analytical approximation matches the real values of n_i well for a wide variety of real and artificial networks. Moreover, we find empirically that the single variable m_i, the embeddedness of node i, is a very good predictor of the percolation probability $\tilde{P}_i$ and thus of the expected audience size. We define the embeddedness of a node as the average embeddedness of all edges attached to that node:

$$m_i = \frac{1}{k_i} \sum_{j \in \mathcal{N}(i)} m_{ij} \ .$$

We also show below the identity $m_i = (k_i - 1)c_i$. Universally, knowing only the degree and clustering (and thus the embeddedness) of a node allows us to make accurate predictions about its expected audience size n_i.

In Extended Data Fig. 4 we illustrate the dependence of n_i on the neighborhood structure of i. Neighborhoods that are highly connected generate the highest expected audiences, while neighborhoods consisting of several isolated individuals create low audiences. Finally, in Extended Data Fig. 5, we show real world networks, where the nodes are colored by their values of n_i. Individuals with many neighbors that are all densely connected among each other have the largest values of n_i. Moreover, for those nodes, the power of indirect reciprocity for incentivizing global cooperation grows strongly with the probability p of gossip transmission.

Relating the embeddedness m_i to the degree and clustering Consider a node i with local clustering coefficient c_i, degree k_i and its neighborhood $\mathcal{N}(i)$. Then

$$m_i = \frac{1}{|\mathcal{N}(i)|} \sum_{j \in \mathcal{N}(i)} m_{ij} = \frac{1}{k_i} \sum_{j \in \mathcal{N}(i)} m_{ij}$$

now let $e_{jl} = 1$ if an edge exists between node j and node l and $e_{jl} = 0$ otherwise ($e_{jj} = 0$). Then $m_{ij} = \sum_{l \in \mathcal{N}(i)} e_{jl}$

$$m_i = \frac{1}{k_i} \sum_{j \in \mathcal{N}(i)} \sum_{l \in \mathcal{N}(i)} e_{jl} = \frac{1}{k_i} \sum_{j,l \in \mathcal{N}(i), j \neq l} e_{jl} \equiv \frac{1}{k_i} k_i (k_i - 1) c_i = (k_i - 1) c_i$$

In the third equality we have used the fact that the expression $\frac{1}{k_i} \sum_{j,l \in \mathcal{N}(i), j \neq l} e_{jl}$ is simply twice (due to double counting jl and lj) the number of edges that exist between all neighbors of i. There are $\frac{1}{2} k_i (k_i - 1)$ possible edges, and the fraction that do exist is given by definition by the local clustering coefficient c_i. Thus, the number that do exist is $\frac{c_i}{2} k_i (k_i - 1)$. Multiplying with the factor of 2 due to double counting we obtain the above result.

Symmetry of n_{ij}

The definition of n_{ij} is fundamentally asymmetric in i and j. However, as shown in the main text, the main first-order dependence of n_{ij} for small p is on the number of mutual neighbors between i and j, which is a symmetric quantity. Therefore, while network growth could arguably depend on a symmetric process of link formation that requires "consent" from both parties, we do not in this work consider alternative symmetric definitions of n_{ij} for the numerical experiments for generating networks, as any symmetric definition capturing information flow on the local friendship network would lead to qualitatively similar behavior.

Subtleties of moral judgment

We assume that every defection carries universally negative reputation, while a cooperation always carries positive reputation. Here we do not consider further "moral" subtleties that arise in judging the reputation value of actions [6, 51], such as whether defecting on a known defector is good or bad [9, 10]. We also do not consider the ability of actors to make strategic decisions of which information to pass on. While these questions are important, our goal is to isolate the effect of social network structure on the spread of reputation. Thus, we choose the simplest model that captures these effects while deliberately not attempting to include the impact of moral judgment.

Transmission of negative information

In our model we only consider the transmission of distinctly positive information. One might also imagine a model where reputation spread can transmit distinctly negative information that would cause a greater punishment [52] or smaller payoff for that node in the future if neighbors find out. Either way, the size of the expected audience determines the strength to be cooperative or to not act negatively.

Other connection incentives

When considering trust based attachment, we have also assumed that considerations of future costs and benefits capture all relevant tradeoffs in forming, keeping, and breaking ties to other nodes. Naturally, there might be other reasons such as bridging structural holes, popularity, or similarity [30, 31, 53, 54] that can modify an individual's incentives for attachment on real social networks. Again, our model aims to isolate the effect of reputation and thus deliberately ignores these other incentives.

Network generation

Picking proportional to m_{ij} and picking random neighbor's neighbor is equivalent

Assume we have a node j and pick a random neighbor's neighbor i. Consider a given such node i. There are then m_{ij} possible paths of arriving at that node i (one for every common neighbor of i and j). Each of these paths has a specific probability of being chosen. The probability of ending up at i is the sum of these "weights" of the various paths. Each path's weight (i.e. its probability of being chosen) depends on the degree of the common neighbor. However, if we assume that degree correlations on the graph aren't too strong, then all paths have in expectation the same weight, independently of the degree of j. Thus, in expectation, the probability of being picked is simply proportional to the number of such paths, n_{ij}.

We show this quantitatively. What is the probability of picking node i via an intermediary node l? We will assume that the nearest neighbor degree distribution on our graph is approximately $P_{nn}(k) = \frac{k_l P(k_l)}{\bar{k}}$. Thus, this distribution is the same as the overall degree distribution, but each degree is upweighted by a factor of k. This is the default for graphs without degree correlations and emerges from the intuition that higher degree nodes have k times more edges that one could be attached to. We have checked numerically that the graphs generated according to our model do not have strong degree correlations. $\bar{k}$ is the average degree on the graph. We will condition on the degree k_l of node l and sum over all possibilities. Recall that node l is picked as a random neighbor of node j, and then i is picked as a random neighbor of l.

$$
\begin{aligned}
P(\text{pick } i \text{ via } l) &= \sum_{k_l} P(\text{pick } i \text{ via } l, k_l) \\
&= \sum_{k_l} P(\text{pick } i \text{ via } l | k_l) P_{nn}(k_l) \\
&= \sum_{k_l} \frac{1}{k_j} \frac{1}{k_l} \frac{k_l P(k_l)}{\bar{k}} \\
&= \frac{1}{k_j \bar{k}} \sum_{k_l} P(k_l) \\
&= \frac{1}{k_j \bar{k}}
\end{aligned}
$$

In the third line, the first term is the probability of picking a given neighbor of j (of which there are k_j). The second is the probability of picking a given neighbor of l. $P(k_l)$ is the degree distribution evaluated at k_l. Thus, each of the m_{ij} paths to node i has equal probability (independently of i), so the total weight is proportional to m_{ij}. Thus, picking random neighbor's neighbors is equivalent to picking a given node with probability proportional to m_{ij}.

Due to its symmetry, the method also leads to two-way trusting relationships, since the methods forms new edges ij proportional to m_{ij}, which is the number of mutual neighbors and thus symmetric, ensuring trustworthiness in both ways.

Why friend-of-friend generates a power law degree distribution

Consider the distribution of degrees among the nodes that are being picked. Regardless of the degree of the intermediate node l, the degree of i is drawn also from the nearest neighbor distribution $P_{nn}(k_i) = \frac{k_k P(k_i)}{\bar{k}}$. Compare this to the

Barabasi-Albert model, where we pick a potential neighbor as a random node from the overall graph (which has degree distribution $P(k)$), with probability proportional to its degree. Thus, picking a node with degree k has probability proportional to $P(k)k$. This is exactly the nearest neighbor degree distribution. Thus, the degree of the new neighbor in friend-of-friend attachment is drawn from the same distribution as in the BA model. We therefore expect to see the same scale free degree distribution overall, while the friend-of-a-friend strategy additionally gives rise to high clustering and embeddedness. The power law emerges naturally from the bias of the nearest neighbor degree distribution and does not require assumptions of popularity or preferential attachment to popular nodes.

Departure of TBA and friend-of-friend for large p

As shown above, the statistical equivalence between TBA and friend-of-friend attachment is only exact in the limit as $p \to 0$. In practice, we find (see Extended Data Fig. 6) that the two methods generate networks with very similar statistics up to moderate values of $p \lesssim 0.25$. For larger values, TBA generates degree distributions with departures from the power law form in the tail of the distribution (even more hubs are generated, which also results in even slower growth of the mean path length). The fact that real life networks do follow power law distributions may suggest that they are generated not directly by TBA, but rather by a heuristic of TBA like friend-of-friend attachment.

Extended data figures

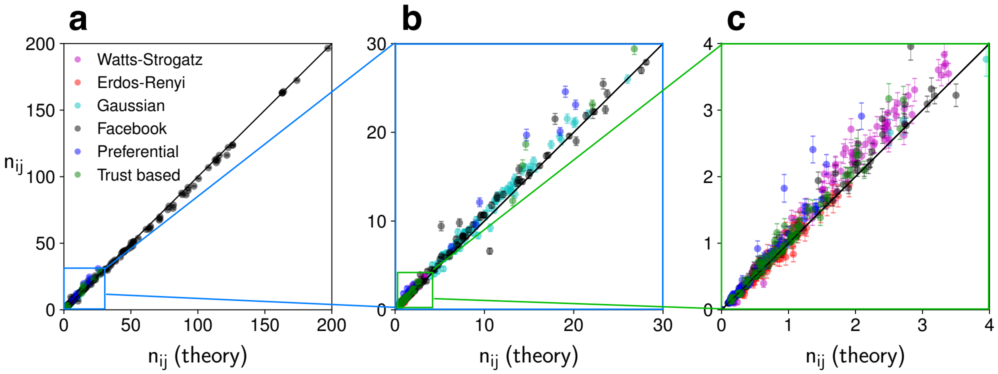

Extended Data Figure 1. Analytical approximation for calculating n_{ij}. We show the numerically computed exact values of n_{ij} vs. our theoretical approximation based on k_i, c_i and m_{ij} (see the section "Random neighborhood approximation for n_{ij}" in the **SI** for details). Each point represents one randomly selected actor and recipient pair, and the pairs are sampled randomly from various artificial and real-world networks. The diagonal solid black line indicates equality. The different panels **(a-c)** zoom into the same plot at various levels of detail. Error bars (s.d.) resulting from the numerical evaluation of n_{ij} are given for each data point. The approximation is universally applicable across various networks sizes and structures. We use 2000 samples for the Monte-Carlo computation of the exact values of n_{ij}. Network parameters — "Watts-Strogatz": small world network [28] with $N = 200$ individuals and an average degree of $k = 20$; "Erdos-Renyi": erdos-renyi random graph with $N = 200$ and $k = 30$; "Gaussian": random 2D geometric random graph with a gaussian connection probability with $N = 200$ and $k = 40$, "Facebook": Facebook social network [39, 42] with $N = 4039$ and $k = 44$; "Preferential": Network generated by preferential attachment [30]; "Trust based": Network generated by trust based attachment (see main text) with $N = 200$ and $k = 20$; $p = 0.1$. We plot 80 points for each graph, except the Facebook graph for which we plot 200 points.

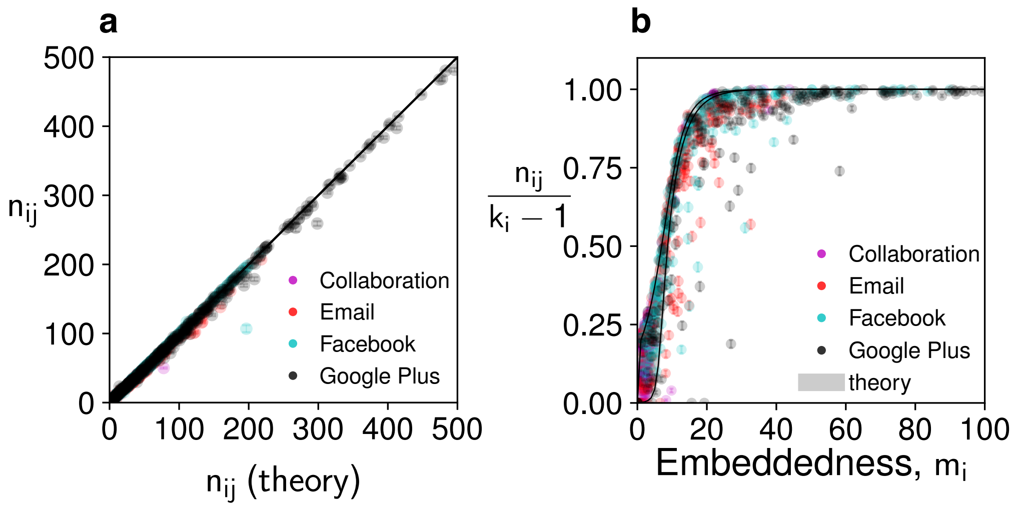

Extended Data Figure 2. Analytical approximation for calculating n_{ij} on real networks. a, Just as for the artificial networks in Extended Data Fig. 1, our full analytical prediction based on k_i, c_i, and m_{ij} (the embeddedness of the edge ij) agrees well with observed values on real networks ([39, 42, 43], see "Size of the expected audience" in the **SI** for details). **b,** As a function of the embeddedness of the actor, the values of n_{ij} approximately collapse onto a narrow range, close to a univariate dependence. We also show how this near collapse as a function of m_i alone is also predicted by our analytical approximation for n_{ij}. In grey, we show the ranges of n_{ij} predicted by our theory constrained by m_i only, obtained by sweeping over possible values of $k_i \in (m_i + 1, N - 1)$ for each m_i, fixing the resulting value of c_i using the identity $m_i = (k_i - 1)c_i$, and using our analytical prediction without using knowledge of m_{ij} (see the **SI** section "Random neighborhood approximation for gossip propagation" for details). In both plots, error bars are shown for each point, which are in most cases smaller than the plotted points. The points are for the same edges and the same graphs as shown in Fig. 2 in the main text. Per graph, 500 randomly selected values of n_{ij} are shown. Parameters: $p = 0.2$.

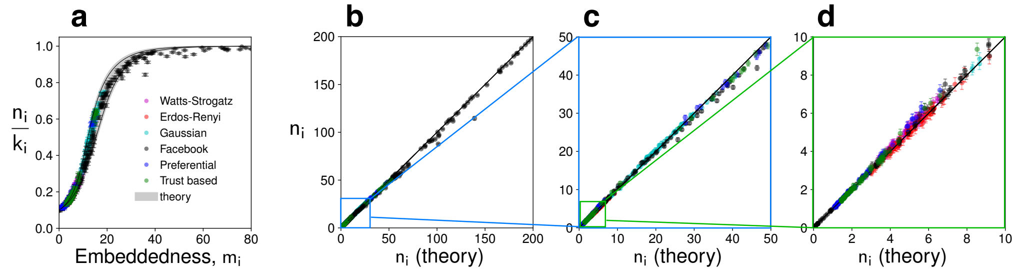

Extended Data Figure 3. Analytical approximation for calculating n_i, the expected audience size for global cooperation. We show the numerically computed exact values of n_i vs. our theoretical approximation based on k_i and c_i (see the section "Global cooperation" in the **SI** for details). Each point represents one randomly selected actor i sampled randomly from various artificial and real-world networks (the same networks as in Extended Data Fig. 2). **(a)** The dependence of the relative audience size $\frac{n_i}{k_i}$ is empirically found to be very well approximated by the single parameter m_i, the embeddedness of node i, which can be written as $m_i = (k_i - 1)c_i$ (see "Global cooperation" in the **SI**). Both the theoretically predicted values (grey region) as well as the actual values of n_i nearly collapse onto a line as a function of m_i. The diagonal solid black line indicates equality. The different panels **(b-d)** illustrate the agreement between the analytical approximation and actual values of n_i. They zoom into the same plot at various levels of detail. Error bars (s.d.) resulting from the numerical evaluation of n_i are given for each data point. The approximation is universally applicable across various networks sizes and structures. We use 2000 samples for the Monte-Carlo computation of the exact values of n_i. Network and plotting parameters are the same as in Extended Data Fig. 1.

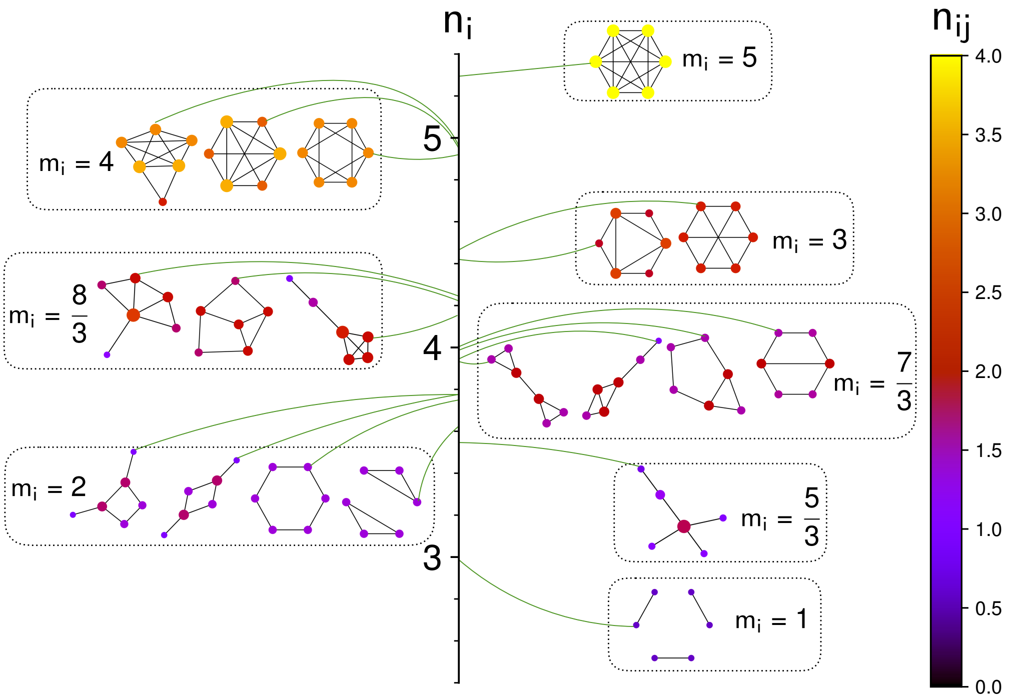

Extended Data Figure 4. Dependence of n_i on neighborhood structure for various neighborhoods of size 6. Each graph represents the neighborhood structure of an actor node i. The actor itself is not shown for clarity. Each node is colored by its value of n_{ij} that the actor has with respect to that node. The entire neighborhood is connected with a thin green line to the value of n_i that the actor has for that neighborhood. The neighborhoods are grouped by the embeddedness m_i, which is highly predictive of the expected audience size n_i. Densely connected neighborhoods create a high expected audience for global cooperation n_i, and centrally connected individuals in the neighborhood enjoy a high expected audience size n_{ij}. Sparsely connected and disjoint neighborhoods have low values of n_i and isolated neighbors have low values of n_{ij}. Parameters: $p = 0.4$.

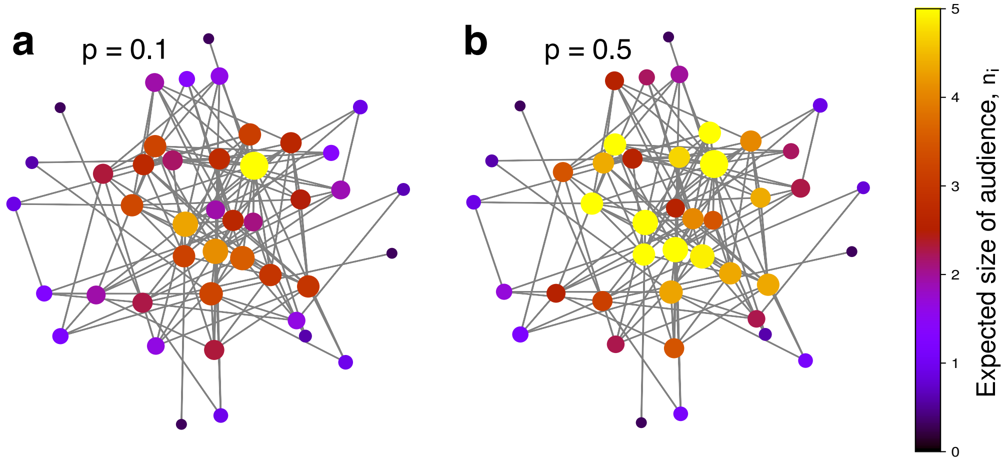

Extended Data Figure 5. Illustration of n_i for a real social network. The network is a subsample of the "Facebook" social network [39, 42]. Nodes are colored by the expected audience size for global cooperation, n_i. The two panels show the same network, but for low **(a)** and high **(b)** values of p. This value is a measure of the incentive for acts of global cooperation (see the section "Global cooperation" in the **SI**). Nodes that are embedded in densely connected, large neighborhoods have high expected audience sizes. Moreover, their incentives grow strongly if the strength of gossip propagation p increases. By contrast, isolated or weakly connected nodes have low values of n_i and increased information flow does not increase their incentives much. Parameters: $N = 40$, $k = 6$, $p = 0.3$.

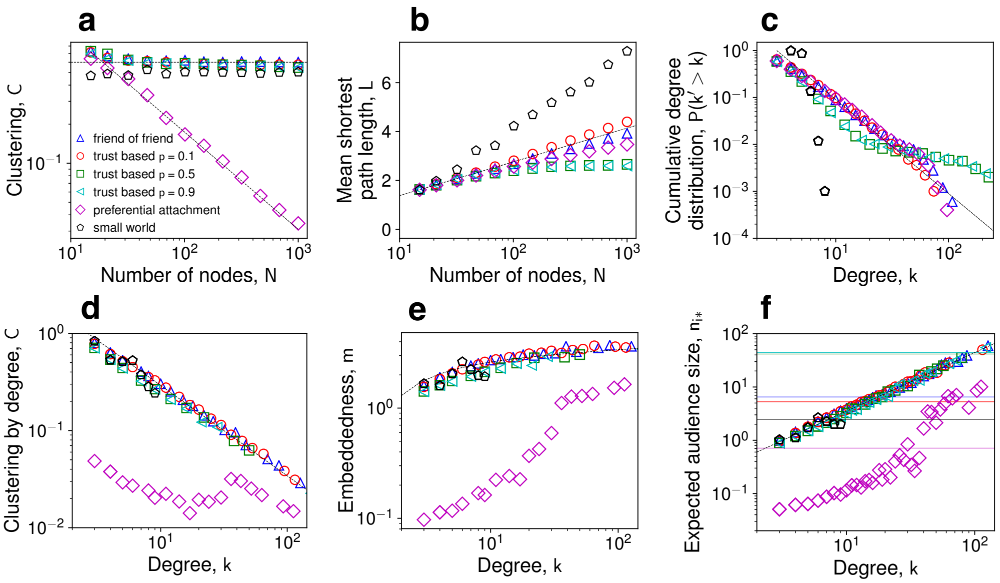

Extended Data Figure 6. Further characterization of graphs generated by trust based attachment. (a-c) The same statistics as shown in **Figure 3**, but also for TBA graphs with $p \in \{0.5, 0.9\}$, as well as small world and preferential attachment networks. TBA with high values of $p \gtrsim 0.2$ shows departures from the friend-of-friend behavior. The degree distribution includes more hubs with even larger degree, and the resulting mean shortest path lengths grow sub-logarithmically with N. Clustering is nearly unchanged.. Clustering is high and decreases as a power law with slope $\sim -0.92 > -1$ (dashed line) for trust based attachment **(d)**. Clustering is consistently low and decreasing with k for preferential attachment. **(e)** The average embeddedness $n = c(k-1)$ — the key indicator for trustworthiness — scales as $c(k)(k-1) \sim k^{-0.92}(k-1)$ (dashed line) and thus grows for higher values of k. **(f)** The mean outgoing expected audience size n_{i*} ($p = 0.5$) scales as a power law with slope ~ 1.1 (dashed line) as a function of k for the TBA and friend of friend networks. The small world network has a near uniform distribution and the preferential attachment network has significantly lower values of n_{i*}. The mean value (averaged over all directed edges) for each network is shown as a horizontal line of the equivalent color. The TBA and friend of friend networks lead to the highest average values of n_{i*}. We find that while these results qualitatively are independent of p, the slope of the power law and the global mean value increase with p. All networks have final size $N = 1000$ and $k = 6$.

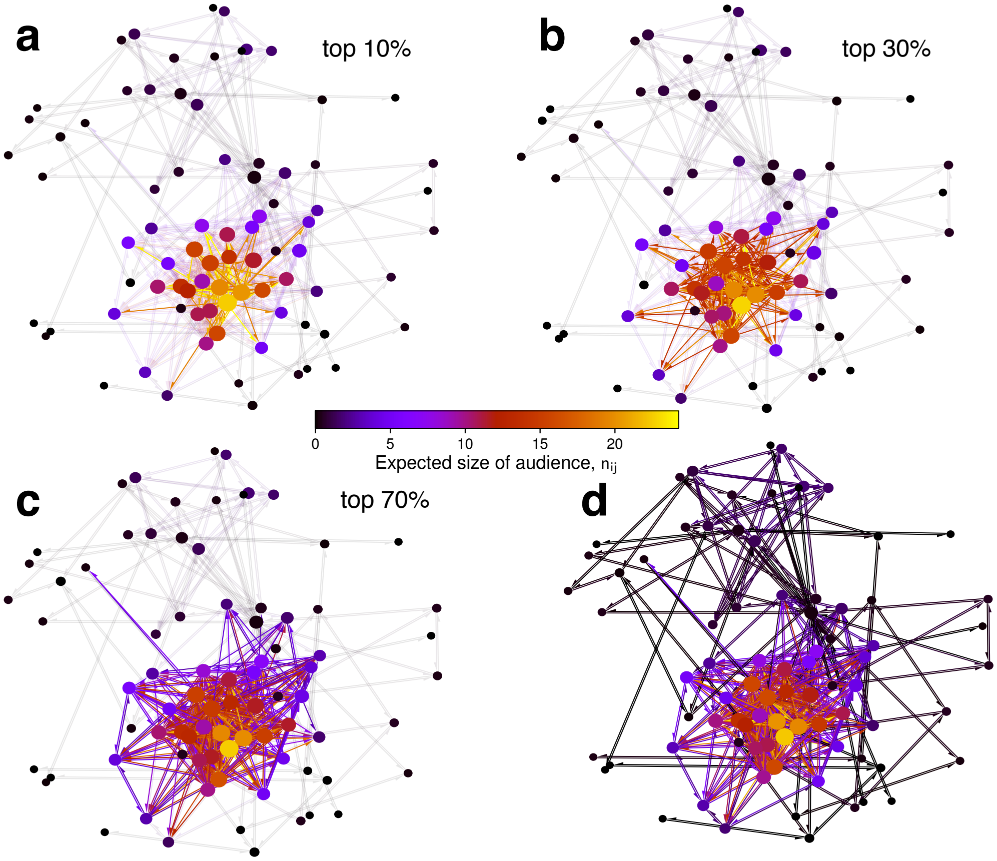

Extended Data Figure 7. Illustration of the hierarchy of n_{ij} values on a real social network. The network is a subsample of the "Facebook" social network ([39, 42] see "Plotting social networks" in the **SI** for details). Edges and nodes are colored as in **Figure 4**. The panels **(a-d)** show progressively larger fractions of the highest n_{ij} values on the network. The highest values occur in densely connected communities, while the lower values occur in individuals that are less embedded and have fewer neighbors. $N = 50$, $k = 6$, $p = 0.4$.

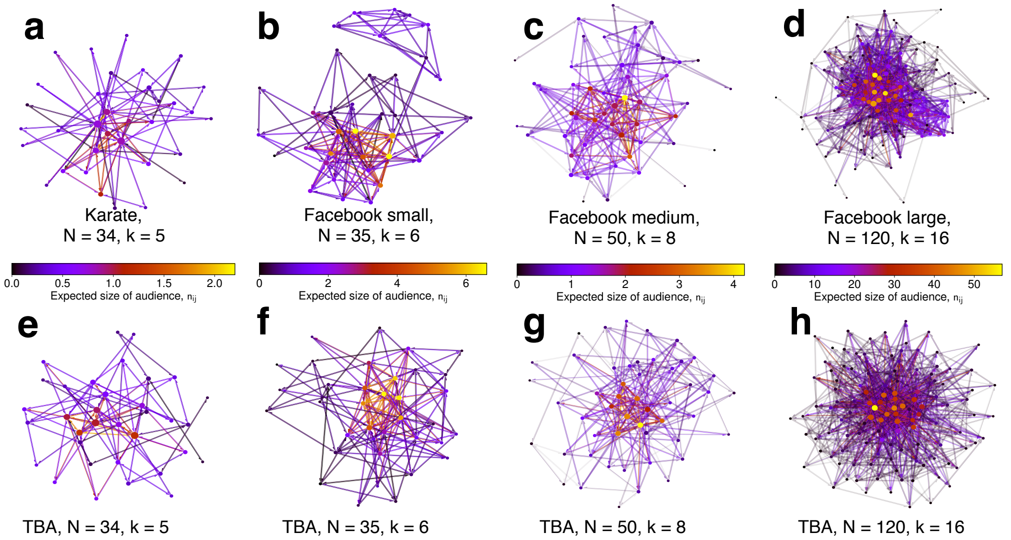

Extended Data Figure 8. Further examples of real and TBA generated networks. The upper row shows various real-world networks (subsamples of the "Facebook" network, [39, 42] see "Plotting social networks" **SI** for details), while the lower row shows networks generated by TBA with the same number of individuals N and average degree k. Edges and nodes are colored as in **Figure 4**. The real-world social networks and the TBA networks both show centrally and highly connected hubs, high clustering, as well as strong incentives throughout. The trust based attachment process generates highly cooperative populations that resemble real-world social networks in key ways.

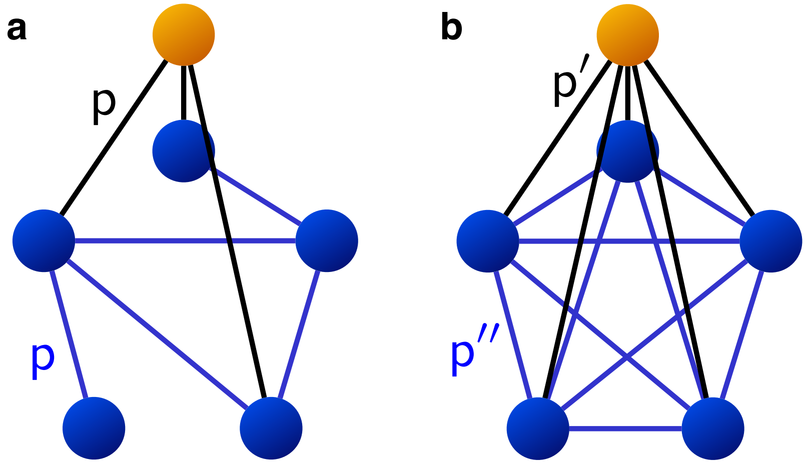

Extended Data Figure 9. Random local neighborhood approximation. We show the actual local neighborhood **(a)** of an actor node i with $k = 6$ neighbors, where the recipient is shown in orange, and the other neighbors in blue. The actor itself is not shown. Every edge can transmit information with probability p. This is equivalent to keeping each edge with probability p and transmitting information across the resulting network. The random graph approximation **(b)** that these edges are randomly distributed. While this is true in expectation, it neglects the real structure of the local neighborhood. Mathematically, the assumption is that all edges from the recipient to the other neighbors (black edges) and all edges among the other neighbors (blue edges) exist at first. They are then kept with modified probabilities such that in expectation, the same number of edges from each category remains for information transfer as in the original graph. The sampling probability p is modified for the black edges (p') and the blue edges (p'') separately. In this case, we would have $p' = \frac{3}{5}p$ and $p'' = \frac{5}{10}p$, since on the real graph **(a)**, only 3 out of 5 possible black edges exist, and 5 out of 10 possible blue edges. See the section "Random neighborhood approximation for gossip propagation" in the **SI** for details.

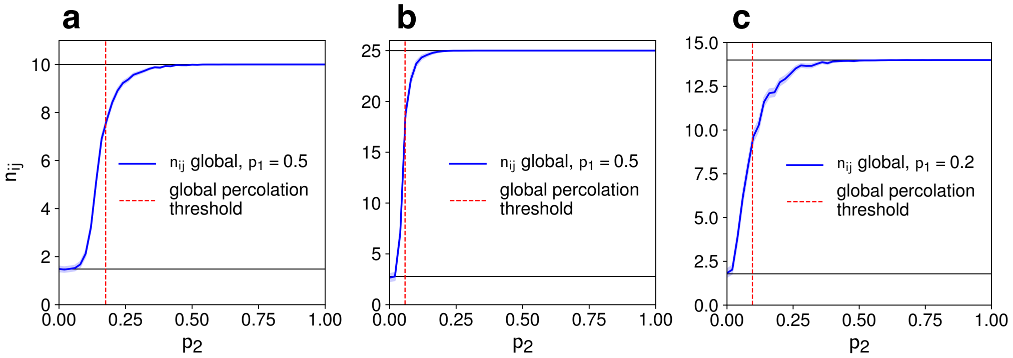

Extended Data Figure 10. Global percolation model. We explore numerically the consequences of a model that allows for information flow on every edge of the actor's neighborhood network with probability p_1, and with probability p_2 on the rest of the network (excluding the edges originating from the actor themselves). In all panels, the value of n_{ij} for a given actor and recipient resulting from this model is shown in blue, where the blue shaded region shows the numerical error (s.d.). The black horizontal lines give the value of n_{ij} for the local model (lower line) and the maximum attainable value of $k-1$ (upper line). The vertical dashed line is the numerically determined edge percolation threshold on the overall network. **(a)** A random actor and recipient are chosen from an Erdos-Renyi network of $N = 400$ nodes with an average degree of 10. **(b)** Same as **(a)** but $N = 1000$ and an average degree of 30. The larger, denser network leads to a sharper percolation transition. **(c)** A random actor and recipient from a social network with $N = 4039$ individuals and an average degree of 44. In all cases, the global percolation threshold p_c determines the key regimes. For $p_2 < p_c$, the value of n_{ij} approaches the value in the local model. For $p_2 > p_c$, n_{ij} approaches $k-1$. For $p_2 \approx p_c$, there is a transition regime whose sharpness and exact behavior is determined by the overall network.

References

1. Alexander R. The biology of moral systems (foundations of human behavior). 1987;.

2. Chalub FA, Santos FC, Pacheco JM. The evolution of norms. Journal of theoretical biology. 2006;241(2):233–240.

3. Wedekind C, Milinski M. Cooperation through image scoring in humans. Science. 2000;288(5467):850–852.

4. Sigmund K. The calculus of selfishness. Princeton University Press; 2010.

5. Engelmann D, Fischbacher U. Indirect reciprocity and strategic reputation building in an experimental helping game. Games and Economic Behavior. 2009;67(2):399–407.

6. Santos FP, Santos FC, Pacheco JM. Social norm complexity and past reputations in the evolution of cooperation. Nature. 2018;555(7695):242.

7. Nowak MA, Sigmund K. Evolution of indirect reciprocity by image scoring. Nature. 1998;393(6685):573–577.

8. Brandt H, Sigmund K. Indirect reciprocity, image scoring, and moral hazard. Proceedings of the National Academy of Sciences. 2005;102(7):2666–2670.

9. Nowak MA, Sigmund K. Evolution of indirect reciprocity. Nature. 2005;437(7063):1291–1298.

10. Ohtsuki H, Iwasa Y. How should we define goodness?—reputation dynamics in indirect reciprocity. Journal of Theoretical Biology. 2004;231(1):107–120.

11. Berger U. Learning to cooperate via indirect reciprocity. Games and Economic Behavior. 2011;72(1):30–37.

12. Radzvilavicius A, Stewart A, Plotkin JB. Evolution of empathetic moral evaluation. bioRxiv. 2018; p. 447151.

13. Bakshy E, Rosenn I, Marlow C, Adamic L. The role of social networks in information diffusion. In: Proceedings of the 21st international conference on World Wide Web. ACM; 2012. p. 519–528.

14. Montanari A, Saberi A. The spread of innovations in social networks. Proceedings of the National Academy of Sciences. 2010;107(47):20196–20201.

15. Centola D. How behavior spreads: The science of complex contagions. vol. 3. Princeton University Press; 2018.

16. Wu X, Liu Z. How community structure influences epidemic spread in social networks. Physica A: Statistical Mechanics and its Applications. 2008;387(2):623–630.

17. House T, Keeling MJ. Insights from unifying modern approximations to infections on networks. Journal of The Royal Society Interface. 2011;8(54):67–73.

18. Borge-Holthoefer J, Baños RA, González-Bailón S, Moreno Y. Cascading behaviour in complex socio-technical networks. Journal of Complex Networks. 2013;1(1):3–24.

19. Watts DJ. A simple model of global cascades on random networks. Proceedings of the National Academy of Sciences. 2002;99(9):5766–5771.

20. Lind PG, da Silva LR, Andrade JS, Herrmann HJ. Spreading gossip in social networks. Phys Rev E. 2007;76:036117. doi:10.1103/PhysRevE.76.036117.

21. Johansson T. Gossip spread in social network Models. Physica A: Statistical Mechanics and its Applications. 2017;471:126–134.

22. Shaw AK, Tsvetkova M, Daneshvar R. The effect of gossip on social networks. Complexity. 2011;16(4):39–47.

23. Pujol JM, Flache A, Delgado J, Sangüesa R. How can social networks ever become complex? Modelling the emergence of complex networks from local social exchanges. Journal of Artificial Societies and Social Simulation. 2005;8(4).

24. Vega-Redondo F. Building up social capital in a changing world. Journal of Economic Dynamics and Control. 2006;30(11):2305–2338.

25. Traag VA, Van Dooren P, Nesterov Y. Indirect reciprocity through gossiping can lead to cooperative clusters. In: Artificial Life (ALIFE), 2011 IEEE Symposium on. IEEE; 2011. p. 154–161.

26. Corten R. Computational Approaches to Studying the Co-evolution of Networks and Behavior in Social Dilemmas. John Wiley & Sons; 2014.

27. Orsini C, Dankulov MM, Colomer-de Simón P, Jamakovic A, Mahadevan P, Vahdat A, et al. Quantifying randomness in real networks. Nature communications. 2015;6:8627.

28. Watts DJ, Strogatz SH. Collective dynamics of 'small-world' networks. Nature. 1998;393(6684):440–442.

29. Kolda TG, Pinar A, Plantenga T, Seshadhri C. A scalable generative graph model with community structure. SIAM Journal on Scientific Computing. 2014;36(5):C424–C452.

30. Barabási AL, Albert R. Emergence of scaling in random networks. Science. 1999;286(5439):509–512.

31. Papadopoulos F, Kitsak M, Serrano MÁ, Boguná M, Krioukov D. Popularity versus similarity in growing networks. Nature. 2012;489(7417):537–540.

32. Bhat U, Krapivsky PL, Redner S. Emergence of clustering in an acquaintance model without homophily. Journal of Statistical Mechanics: Theory and Experiment. 2014;2014(11):P11035.

33. Davidsen J, Ebel H, Bornholdt S. Emergence of a small world from local interactions: Modeling acquaintance networks. Physical Review Letters. 2002;88(12):128701.

34. Leskovec J, Kleinberg J, Faloutsos C. Graphs over time: densification laws, shrinking diameters and possible explanations. In: Proceedings of the eleventh ACM SIGKDD international conference on Knowledge discovery in data mining. ACM; 2005. p. 177–187.

35. Wu Z, Menichetti G, Rahmede C, Bianconi G. Emergent complex network geometry. Scientific reports. 2015;5.

36. Bhat U, Krapivsky P, Lambiotte R, Redner S. Densification and structural transitions in networks that grow by node copying. Physical Review E. 2016;94(6):062302.

37. Bianconi G, Darst RK, Iacovacci J, Fortunato S. Triadic closure as a basic generating mechanism of communities in complex networks. Physical Review E. 2014;90(4):042806.

38. Lambiotte R, Krapivsky P, Bhat U, Redner S. Structural Transitions in Densifying Networks. Physical review letters. 2016;117(21):218301.

39. Leskovec J, Krevl A. SNAP Datasets: Stanford Large Network Dataset Collection; 2014. `http://snap.stanford.edu/data`.

40. Rand DG, Arbesman S, Christakis NA. Dynamic social networks promote cooperation in experiments with humans. Proceedings of the National Academy of Sciences. 2011;108(48):19193–19198.

41. Cuesta JA, Gracia-Lázaro C, Ferrer A, Moreno Y, Sánchez A. Reputation drives cooperative behaviour and network formation in human groups. Scientific reports. 2015;5:7843.

42. Leskovec J, Mcauley J. Learning to discover social circles in ego networks. Advances in neural information processing systems. 2012;25.

43. Leskovec J, Kleinberg J, Faloutsos C. Graph evolution: Densification and shrinking diameters. ACM transactions on Knowledge Discovery from Data (TKDD). 2007;1(1):2–es.

44. Raghavan UN, Albert R, Kumara S. Near linear time algorithm to detect community structures in large-scale networks. Physical review E. 2007;76(3):036106.

45. Kirkpatrick S. Percolation and conduction. Reviews of modern physics. 1973;45(4):574.

46. Callaway DS, Newman MEJ, Strogatz SH, Watts DJ. Network Robustness and Fragility: Percolation on Random Graphs. Phys Rev Lett. 2000;85:5468–5471. doi:10.1103/PhysRevLett.85.5468.

47. Masum H, Tovey M, Newmark C. The reputation society: How online opinions are reshaping the offline world. MIT Press; 2012.

48. Easley D, Kleinberg J. Networks, crowds, and markets. Cambridge Univ Press. 2010;6(1):1–6.

49. Gilbert EN. Random graphs. The Annals of Mathematical Statistics. 1959;30(4):1141–1144.

50. Serrano MÁ, Boguná M. Clustering in complex networks. II. Percolation properties. Physical Review E. 2006;74(5):056115.

51. Hilbe C, Schmid L, Tkadlec J, Chatterjee K, Nowak MA. Indirect reciprocity with private, noisy, and incomplete information. Proceedings of the National Academy of Sciences. 2018;115(48):12241–12246.

52. Jordan JJ, Hoffman M, Bloom P, Rand DG. Third-party punishment as a costly signal of trustworthiness. Nature. 2016;530(7591):473.

53. Burt RS. Structural holes and good ideas. American journal of sociology. 2004;110(2):349–399.

54. Pentland A. Social physics: How good ideas spread-the lessons from a new science. Penguin; 2014.
